# Utilisation of healthcare by immigrant adults relative to the host population: Evidence from Ireland

**DOI:** 10.1016/j.jmh.2021.100076

**Published:** 2021-11-26

**Authors:** Peter Barlow, Gretta Mohan, Anne Nolan

**Affiliations:** a Economic and Social Research Institute, Dublin, Ireland; b Department of Economics, Trinity College, Dublin, Ireland; c The Irish Longitudinal Study on Ageing, Trinity College, Dublin, Ireland

**Keywords:** Migrant, Healthcare, General Practitioner utilisation, Consultant utilisation, Health economics

## Abstract

**Objective:**

While there is a broad consensus that barriers to access in the utilisation of healthcare exist for immigrants in the US, European evidence exploring this issue paints a mixed picture, with studies from a variety of European jurisdictions presenting different conclusions. In this context, Ireland, a European country with substantial private involvement in healthcare delivery, and, a largely young immigrant population, provides an opportunity to investigate the healthcare utilisation of immigrants compared to natives in a European country with mixed private-public healthcare provision.

**Design:**

The healthcare utilisation patterns of immigrants (defined as residents with a foreign country of birth) and native-born participants were analysed from a nationally representative health survey of 6,326 adults, carried out in Ireland in 2016. An array of socio-economic and health information was collected such that regression analysis on healthcare consultations accounted for confounding factors.

**Results:**

Non-native residents of Ireland born outside the UK were less likely to have attended a General Practitioner (Odds ratio (OR): 0.62 [95% Confidence Interval (CI): 0.51–0.74]; *p*<0.001) or consultant doctor (OR: 0.60 [95% CI: 0.47–0.76]; *p*<0.001) in the previous year, relative to Irish-born individuals. UK-born residents of Ireland displayed similar utilisation patterns to those of the native population in terms of GP visitation, but a higher likelihood of having attended a consultant (OR: 1.44 [95% CI: 1.14–1.816]; *p* = 0.004).

**Conclusions:**

Lower use of healthcare by those born outside Ireland and the UK relative to the native Irish population may be due to different approaches to healthcare utilisation or obstacles to healthcare utilisation. The findings suggest that the utilisation of healthcare by immigrants merits continued policy attention to respond to the needs of these key groups in society and facilitate integration.

## Highlights

• Non-UK migrants in Ireland were less likely to visit a GP than those local-born.
• Non-UK migrants in Ireland were less likely to attend a consultant.
• Different migrant groups had different patterns of healthcare use.
• Healthcare use by immigrants is context specific.

## 1 Introduction

The use of healthcare by migrants has been a topic of considerable discussion in both the public sphere and in academic literature. We note that there is no consensus on a definition of a ‘migrant’- which can be defined differentially according to foreign birth, foreign citizenship and/or duration of stay in a host country (Anderson and Blinder 2011). Additionally, the terms ‘migrant’ and ‘immigrant’ are often used interchangeably; an ‘immigrant’ may be understood as a person who is, or intends to be, settled in a new country, while a ‘migrant’ may be temporarily resident in the new country. From a healthcare provision perspective, those who move to a new country have been identified as a “vulnerable population”, with an acute risk of problems relating to their mental or physical health (Derose et al., 2007, p. 1258). While barriers to the utilisation of healthcare services by migrants have been widely documented within the context of the US market insurance-based healthcare system, the evidence concerning the use of healthcare by migrants in European countries demonstrates more nuanced patterns across nations and types of healthcare services.

The empirical study of patterns of healthcare utilisation by county of birth is important because immigrants represent a key demographic for developed, but ageing societies. There are currently over 1 billion migrants in the world today, with the migrant population accounting for 10% of the population of the World Health Organization (WHO) Europe region (World Health Organization 2018). International migration is expected to continue to grow, with the US projected to see its migrant population expand by 25% between 2016 and 2060 according to the US Census Bureau (Johnson 2020). The EU projects that net immigration will reach 66.2 million between 2016 and 2080 (Eurostat 2019). In Ireland, the migrant population is expected to increase by 10,000 per year until 2051 under the most conservative demographic change scenario (CSO 2020). These anticipated trends have important implications for policymaking and planning in healthcare settings. More broadly, accommodating the needs of migrants in terms of healthcare provision may also facilitate greater integration into host societies and address inequalities in health and other areas. The WHO has published a Global Action Plan which sought to target the improvement of health and healthcare provision amongst migrants in which concern was expressed that “migrants lack access to health care services” (World Health Organization 2019, pg. 3).

For the purposes of this paper, the term ‘migrant’ is based on information from where people included in this study report to have been born, however, we acknowledge this definition does not distinguish between many different types of migrants including economic migrants, family reunification migrants, refugees, etc. We seek to examine the effect of having been born in a different country to the host nation on healthcare utilisation. In the context of studies examining access to healthcare, migrant status may influence the “predisposing, enabling and need components of individual accessibility” theorised in the Anderson model of healthcare access (Aday and Andersen 1974, pg 213). A migrant's disposition towards the use of General Practitioner (GP) and hospital services irrespective of illness may differ from native Irish people. For example, culture and health-related behaviours may result in different propensities to use healthcare. Migrants may have differing enabling factors for healthcare access such as financial resources, information available to them and language differences. Migrants may have differing levels of need, due to varying exposures to disease. To date, the international literature has identified significant obstacles in access to healthcare for immigrants, ranging from conventional financial barriers by which immigrants in low skilled professions may be especially vulnerable, to specifically targeted statutory barriers surrounding their immigration status (Derose et al., 2007).

Within Europe, Ireland provides an interesting study setting of migrant healthcare use as the provision and financing of primary healthcare, via consultations with GPs, is largely based on a private free market approach, supplemented by public intervention in the form of a medical card and GP visit card scheme to provide free care for low-income groups (somewhat comparable with the Medicaid system operating in the US). The healthcare arrangements for immigrants residing in Ireland are the same as those for the native population (detailed in Section 2 of this paper).

Moreover, large net inward flows of migration to Ireland is a relatively recent phenomenon. While there were some instances of positive net immigration in the 1970s, the beginning of a more long-term trend for large-scale immigration occurred from 1996 and increased rapidly from 2004 to 2007, attributable to the enlargement of the EU which granted free movement of workers from Accession countries, mainly from Eastern Europe. The Great Recession of 2008/9 resulted in a sharp decline in inward migration to Ireland, but from 2015 net immigration resumed alongside a strong economic recovery. Ireland's immigrant population is characterised by largely young, working age adults.

The most recent Irish Census taken in 2016 revealed that overall, 85% of the resident Irish population was composed of individuals born in Ireland, while 11% of residents were non-nationals and 4% were naturalised immigrants (CSO 2016). Traditionally, the largest proportion of immigrants to Ireland came from the neighbouring UK, as a result of strong familial and employment ties, and a longstanding Common Travel Area (Gilmartin 2013). The 2016 Irish Census showed that individuals of a UK origin accounted for 19% of the non-Irish born resident population (CSO 2016), though this proportion was exceeded by Polish-born residents, who presently make up the largest share of those born outside Ireland (23%). Moreover, Eastern Europeans from Lithuania accounted for 7% of the foreign-born population, while 5% came from Romania and 4% from Latvia (CSO 2016). Other countries also made up a notable share, including Brazilians and Spanish who respectively accounted for 3% and 2% of non-Irish born. We note that this paper is written as the COVID-19 pandemic unfolds, so the full effects of this global crisis on economic conditions and migration flows have yet to be observed.

In recognising the greater representation of migrants and ethnic minorities in the Irish population, the Irish Health Service Executive (HSE) published a *Second National Intercultural Health Strategy* in 2017, outlining a vision for “A health service that empowers service users from diverse ethnic, cultural and religious backgrounds to access services” (HSE 2017, pg. 9). More broadly, the importance of equity of access to healthcare has been underscored in *Sláintecare*, a policy which outlines plans to reform the Irish healthcare system to one which provides universal access (Department of Health 2019a).

The aim of this paper is to empirically investigate the use of doctor-provided healthcare services by adults in Ireland across native and foreign-born backgrounds to discover whether there are differences in utilisation of services across these groups. The research question enquires: Do non-native residents of Ireland use healthcare differently from the local native population?

The outline of this paper is structured in the following manner. The next section explains the set-up of the Irish healthcare sector. Literature concerning the impact of migration status on healthcare access is then explored and interpreted. This is followed by a description of the data and methods employed to carry out the analysis. The results are discussed, with conclusions summarised in the final section.

## 2 Institutional Context

Ireland's healthcare system is a mixture of public and private provision and financing. GPs provide a point of first contact, acting as gatekeepers to specialist care. In Ireland, patients are categorised into a two-tier system for access to public healthcare services: Category 1, full eligibility, who are entitled to a medical card under the General Medical Services (GMS) scheme, or Category 2, limited eligibility.

Category 1 patients hold a medical card that entitles them to free consultations with a GP with whom they are registered. The government reimburses the GP by a capitation system. Individuals on low incomes or for whom illnesses could result in significant financial hardship can apply for a medical card. The entitlements of medical cardholders include free care in public hospitals and significantly reduced co-payments for medicines. In 2018, 32.4% of Irish adults possessed a medical card (Department of Health 2019b). A further 10% held a GP visit card, which like the medical card provides free access to GP care for patients, but in terms of accessing other healthcare services GP visit card holders are considered private patients. The entitlement to the GP visit card is based on a slightly higher income threshold, or automatic entitlement occurs for those aged over 70 years, those under 6-years-old and carers.

Category 2 patients are private patients who must pay for access to GP consultations at the point of service, with an average visitation charge of €52.50 (Connolly et al., 2018). They also pay the full cost of medicines subject to a monthly deductible. Category 2 patients are entitled to free care in public hospitals but are subject to co-payments for Emergency Department (ED) attendances and in-patient nights.

A substantial proportion of people (43%) in Ireland also purchase private insurance (Department of Health 2019b). Private health insurance may be purchased by either Category 1 or Category 2 patients, the main benefit of which is acute hospital care provided by a private provider to circumvent public waiting lists.

While many European countries extend universal primary care to migrant populations, the healthcare arrangements for migrants residing in Ireland are the same as those for the native population. A person living in Ireland for at least one year is considered to be ‘ordinarily’ resident and is entitled to either full eligibility (Category 1) or limited eligibility (Category 2) for public health services. People who have not been resident in Ireland for at least one year must satisfy the HSE that it is their intention to remain for a minimum of one year to be eligible for health services (Cross care, 2019).

## 3 Literature review

There has been considerable discussion of the factors affecting utilisation and access to healthcare for migrants. These often depend on the immigration and healthcare financing structures which exist in a jurisdiction. In an umbrella review of nine systematic reviews of literature related to healthcare utilisation amongst immigrants in developed countries, Gil-González et al. (2015) identified that structural barriers related to possession of health insurance, cost of drugs and system organisation were most often identified. To inform the context of the investigation of this paper, the literature concerning the use of healthcare services by native and migrant populations emanating from the US is first documented, followed by European evidence.

### 3.1 US literature

Systematic reviews of various strands of the literature related to healthcare use in the US document consistent findings of access barriers encountered by a wide variety of different immigrant subgroups. Systematic reviews concerning black immigrants, Asian immigrants and undocumented immigrants to the US have all detected significant difficulties in accessing healthcare for these populations (Clough et al., 2013; Hacker et al., 2015; Wafula and Snipes 2014). The difficulties identified in these reviews included language and cultural barriers, but they also referred to lack of insurance or financial assets.

Ku and Matani (2001) analysed the National Survey of American Families finding that non-citizen immigrants were more likely to lack a usual source of care or health insurance. Mohanty et al. (2005) found that immigrants and particularly marginalised ethnic groups spent less on healthcare than the average American for all age groups. The authors concluded that immigrants, though they experienced significant financial barriers to healthcare such as a lack of insurance, were also found to experience alternative barriers to care such as the impact of welfare reform and a fear of deportation.

The use of healthcare in the US by the Hispanic immigrant population has received considerable scholarly attention. An investigation of immigrant utilisation of healthcare at the US-Mexico border by Wallace et al. (2009) illustrated that Mexican immigrants to the US were more likely to seek medical, dental and prescription services in their home country rather than in the US. Those within 120 miles of the border with Mexico had higher rates of utilisation explained by a preference for utilisation in their home country. Moreover, Hoerster et al. (2010), in a survey of farm labourers in California, found that Hispanic immigrants were less likely to utilise healthcare in the US and less likely to have insurance than US workers. In a study using data from the US National Health Interview Surveys for 2000–2017 in an Andersen framework, Shafeek Amin and Driver (2020) found that Middle Eastern women were more likely to have visited the GP in the previous 12 months than Middle Eastern men. Healthcare utilisation by Hispanic immigrants was also found to be affected by their education and employment status unlike other immigrant groups to the US.

Immigration status was also found to have a significant negative effect on healthcare service utilisation in an analysis of the 2003 California Health Interview Survey (Ortega et al. 2007). While the findings indicated that immigrants utilised EDs and physicians significantly less often, they also found that immigrant authorisation status had a further impact on healthcare utilisation where undocumented immigrants utilised healthcare less than US-born individuals. Similarly, in a study using the Los Angeles Family and Neighbourhood Survey, Goldman et al. (2005) found that foreign born individuals were particularly likely to be uninsured. This was particularly the case amongst the Hispanic immigrant population. However, much of the lack of insurance could be explained from the effects of education and employment status. A residual impact was detected for undocumented immigrants even where these factors were taken into account.

Studies from north of the US border in Canada may also be of interest in the context of the study setting presented here since the Canadian healthcare system is characterised by universal access, as in most of Europe. Comparing primary care access between the US and Canada, Siddiqi et al. (2009) revealed that differences between insured and uninsured immigrants were lower in Canada under universal healthcare relative to the US. In another study of 20 immigrant families to Montreal, Leduc and Proulx (2004) indicated that while healthcare utilisation was dependant on an individual's ethnicity, newly arrived immigrants tended to utilise EDs more often. However, the duration of time spent in the country emerged as important, whereby the longer immigrants were in Canada, the more they used primary care.

### 3.2 European literature

Given the diversity of countries, native cultures and immigrant populations in Europe, a review of the literature for the European context begins with summarising the findings of systematic reviews, followed by an overview of findings of relevant studies from individual European nations. A systematic review which considered healthcare utilisation amongst a range of healthcare services across six European countries (Norredam et al., 2010) identified that most studies concerned with the use of GP care found immigrant utilisation to be higher than that of non-migrants (7 out of 10 studies). A subsequent review of 39 European-based publications suggested a mixed picture on the use of healthcare services by migrants (Graetz, 2017). The authors found it difficult to make firm conclusions on migrant utilisation patterns, explaining that different countries in Europe varied significantly in terms of the migrant group under analysis, particularly regarding the demographics of the groups being compared. For example, the nationalities of the immigrants and their composition in terms of age varied across countries studied. This may be due to historical factors, for example, a common language or links due to historic European colonialism e.g. the UK's Commonwealth citizenship, or active policies by countries such as recruitment agreements e.g. in the 1960s the Netherlands invited workers from Turkey, Morocco and Southern Europe. Moreover, the indications in the results varied across healthcare utilisation types. Immigrants were more often found to have lower rates of GP utilisation while they were more likely to have higher rates of specialist care utilisation. Universal healthcare is typically extended to immigrants due to the nature of European systems (Mladovsky 2009), and thus the discussion around barriers to healthcare focuses less on impediments related to cost or market provision.

A study of Dutch individuals signed up to the public insurance register revealed that people from Suriname, Turkey and Morocco were more likely to use GP services than the Dutch-born population (Stronks et al., 2001). However, they also found that type of healthcare utilisation varied by immigrant group. For example, Turkish and Moroccan individuals had a relatively low use of specialist healthcare services relative to the Dutch, though Surinamese immigrants had similar utilisation of specialist care to the native-born group; and individuals from the Netherlands Antilles used hospital services rather than primary care. Schoevers et al. (2010) revealed that undocumented female immigrants to the Netherlands faced obstacles accessing healthcare facilities and that poor language proficiency amongst this group reduced utilisation of primary healthcare.

A study from England found little evidence of greater levels of utilisation of primary care for immigrants (identified as such because they had registered with the National Health Service after the age of 15 years), instead finding similar rates of attendance to native individuals (Steventon and Bardsley 2011). However, Stagg et al. (2012) found that immigrants to the UK were less likely to be registered with a GP. In terms of hospital care, Livingston et al. (2002) suggested there was higher utilisation of hospital care amongst older immigrants in Islington, London. Though, Cooke et al. (2007) found no difference between immigrant utilisation of hospitals and the general population for an infectious diseases department in London.

Turning to literature from Ireland, the country that is the focus of this paper, Villarroel et al. (2019) undertook a scoping review of studies which had some consideration of migrant health based in Ireland, including 80 studies. Most studies examined the overall *health status* of migrants, with fewer references to *healthcare utilisation*. The lack of quantitative investigations of the use of healthcare in Ireland by migrant populations stood out as a gap in the review. By contrast, some qualitative studies included in the review provided insight to the experiences of migrants navigating the healthcare system in Ireland. In a qualitative analysis of interviews conducted with 60 immigrants, Migge and Gilmartin (2011) found that immigrants had difficulty understanding the complex Irish healthcare system, and had problems adjusting to the quality of care and affording private health insurance. The interviews revealed that barriers to access were prompting patient mobility, where immigrants would travel to their country of origin to get healthcare. Similarly, in a qualitative study based primarily on informal interviews with individuals and non-governmental organisations, Stan (2015) found that Romanian migrants living in Ireland were compensating for low engagement with the Irish healthcare system by greater use of the Romanian healthcare system. An analysis of the differing attitudes towards healthcare between Irish natives and Polish migrants found that Polish migrants were more likely to classify certain levels of health as being a less desirable health state, and were more likely to assign their health a negative value, than those Irish-born (Kelleher et al. 2020). This may imply that Polish immigrants have different health behaviours in response to an illness, and different propensities to use healthcare.

To summarise, studies relating to migrants residing in the US generally conclude that immigrants face significant barriers to accessing healthcare compared to the native population, however, the evidence from the European literature is less definitive. While it does not have the very high level of private financing of healthcare that exists in the US, Ireland does provide an interesting case study of healthcare use in a European context since it has a blended model of public and private financing and provision. Ireland is an outlier in a Europe as its primary care sector is largely based on private financing. The country's relatively recent accommodation of foreign-born populations also provides a distinctive background in which to view the extent of integration and assimilation. To the best of our knowledge, the use of doctor's services by adult immigrants relative to the Irish population has not been quantitatively investigated using a nationally representative survey.

## 4 Materials and methods

### 4.1 Data

#### 4.1.1 Healthy Ireland

The source of data for this analysis is the 2016 *Healthy Ireland* survey, which is an annual face-to-face survey carried out by an external market research company, Ipsos MRBI, on behalf of the Department of Health in Ireland. The objective of the survey is to provide an overview of the health of the population to inform and develop health policy. The sampling frame was the Irish Geodirectory, which records the geographic location of all the addresses in Ireland, and participants were selected randomly. Approximately 7500 interviews were conducted, achieving a 59.9% response rate (Ipsos MRBI 2016a). Information on the locations of GPs in Ireland was also linked to the survey information to account for supply-side considerations.

#### 4.1.2 Outcomes of interest

Two outcome variables are examined, one concerning the use of GP services and another concerning a respondent's contact with a consultant doctor. Survey participants were asked whether they had attended the GP in the previous 12 months, to which they could give a ‘yes’ or ‘no’ response. The dependant variable concerning the use of GP services takes a value of 1 where the respondent answered ‘yes’ to having attended a GP in the previous year, and 0 otherwise. Similarly, a respondent was asked whether they had attended a consultant in the previous 12 months, responding with ‘yes’ or ‘no’. The dependant variable concerning the use of consultant services takes a value of 1 if the respondent answered ‘yes’ to having attended a consultant in the previous year, and 0 otherwise.

#### 4.1.3 Exposure of interest

Immigrant status was determined based on the individual's country of birth. A question asked in which country the individual was born. The respondent stated their native country. For the purposes of this research, three categories of country of birth were assessed:

• Born in Ireland;
• Born in the UK; and
• Born in countries other than Ireland or the UK (Other).

Based on this categorisation, a variable was created indicating whether an individual was native born, born in the UK or born in a country other than Ireland or the UK. In Table 1, we note that 83.5% of the *Healthy Ireland* sample for analysis were born in Ireland, which is similar to the 2016 Irish census, where 85% of the population were native Irish and 11% were born outside Ireland (CSO 2016). However, we note there is an overrepresentation of those born in the UK in our sample (6.1%) compared with the census (2%), most likely due to differences in how *Healthy Ireland* and the census considers country of birth for those from Northern Ireland which is part of the United Kingdom, but on the island of Ireland.^1^

**Table 1:** Summary statistics – Healthy Ireland sample for analysis.

Variable	Category	Sample used for analysis(%)
*GP utilisation*	Attended GP in previous 12 months	75.2
	Did not attend GP in previous 12 months	24.8
*Consultant utilisation*	Attended consultant in previous 12 months	28.8
	Did not attend consultant in previous 12 months	71.2
*Immigrant status*	Irish-bornUK-born immigrantNon-UK born immigrant (born in a country outside Ireland and the UK - Other)	83.56.110.4
*Gender*	Male	46.4
	Female	53.6
*Age class*	15–2425–4445–6465 or greater	7.533.832.925.8
*Marital status*	MarriedUnmarried	56.243.8
*Education*	Primary	10.3
	Secondary	46.5
	Tertiary	43.2
*Social class (manual labourer)*	Yes	33.8
	No	66.2
*Urban*	UrbanRural	61.039.0
*Region*	DublinMunsterNon-Dublin LeinsterConnaught/Ulster	22.226.528.922.4
*Supply of GPs in locality (quintile)*	0.No GP in 1.6 km1.Least GPs in 1.6 km2.3.4.5.Most GPs in 1.6km	36.615.112.111.813.211.3
*Medical card status*	Medical card holder	35.5
	GP visit card holder	6.4
	No medical card or GP visit card	58.1
*Private health insurance status*	Has private health insuranceNo private health insurance	51.148.9
*Self-rated health*	Good or very goodFair, poor or very poor	84.016.0
*Daily smoker*	YesNo	16.084.0
*Long term Illness in past 12 months*	YesNo	29.770.3
*Diabetes*	YesNo	4.895.2
*Arthritis*	YesNo	12.387.7
*High blood pressure*	YesNo	15.584.5
Sample observations		6326

The summary statistics for the original Healthy Ireland sample (i.e. prior to dropping cases that have missing data on variables of interest) is provided in Supplementary File Table A1.

#### 4.1.4 Other covariates

*Healthy Ireland* captures a wide variety of demographic, socio-economic and health related information. This permitted the controlling of important, potentially confounding factors in the study of healthcare utilisation such as gender, age, marital status, educational attainment, social class, urban location, region, medical card status, GP card status, possession of private health insurance, the individuals’ own rating of their health, whether the individual was a daily smoker, whether the individual had an illness in the previous 12 months, whether they had specific conditions such as diabetes, arthritis and high blood pressure. The level of GP supply in the individual's locality as measured by the concentration of GPs within 1.6 km (20-minute walking distance) also permitting controlling for supply-side considerations. Summary statistics for the covariates are shown in Table 1.

### 4.2 Estimation methodology

#### 4.2.1 Logistic regression

A logistic regression model was used to detect variation in the utilisation of healthcare between native born respondents and those from different foreign-born groups. The estimation of the likelihood of an individual having visited the GP or a consultant may be represented as:

(1) $$Pr\left(u_{i}=1\right)=\frac{\text{exp}\left(\beta_{0}X_{0}+\beta_{I}X_{I}+\;\beta_{d}X_{d}\right)}{1+\text{exp}\left(\beta_{0}X_{0}+\beta_{I}X_{I}+\;\beta_{d}X_{d}\right)}\;$$

(2) $$Pr\left(u_{i}=1\right)=\frac{\text{exp}\left(\beta_{0}X_{0}+\beta_{I}X_{I}+\;\beta_{d}X_{d}+\;\beta_{S}X_{S}\right)}{1+\text{exp}\left(\beta_{0}X_{0}+\beta_{I}X_{I}+\;\beta_{d}X_{d}+\;\beta_{S}X_{S}\right)}\;$$

Where *Pr*($u_{i}$ = 1) represents the probability that an individual i visited a GP or consultant in the previous 12 months. This probability is a function of $X_{I}$, which captures the main exposure of interest, immigrant status (country of birth group). In the models above, *exp* denotes that model is an exponential of a value to the power of the variables and their coefficients. For model (1) the basic demographic factors of age and sex are also adjusted for, symbolised by $X_{d}$. Model (2) expands model (1) to adjust for the socio-economic and health circumstances of the individual, represented by $X_{s}$, described in the section concerning covariates (Section 4.1.4).

The effects of the variables $X_{I}$, $X_{d}$ and $X_{s}$ are determined based on their corresponding coefficients, β_I,_ β_d_ and β_s_. These coefficients indicate the effect which the X values have on the probability that an individual attended the GP/consultant. Thus, we can determine the likelihood of an individual having attended the GP/consultant in the past 12 months, expressed as an odds ratio. An odds ratio of greater than 1 can be interpreted as an individual being more likely to have attended the GP/consultant, with 1 representing the likelihood of attending in the reference category (Irish born in this case). The threshold for determining statistical significance is a p-value of *p*<0.05.

We note that the sample used for analysis is that for which there are complete cases. Individuals with missing data across the study variables were dropped from the analysis. As a result, the sample for analysis fell from the original 7498 respondents to 6326. The characteristics of the analytical sample and the original sample are similar, demonstrated in Table 1 and Supplementary File Table A1.

#### 4.2.2 Robustness checks

Several robustness checks were carried out to verify the results of the main analysis. The number of visits to the GP and a consultant doctor in the previous four weeks was also asked in the interview, and this was also examined for the country of birth groups. The models included an OLS regression approach, as well as an ordered logit and a partial proportional odds model to incorporate both the annual and monthly visitation information.

## 5 Results

The summary statistics are presented first, followed by the model estimation results for GP attendances and those for consultants.

### 5.1 Summary statistics

Table 1 shows that 75.2% of the analytical sample visited the GP in the previous 12 months, while 28.8% had attended a consultant doctor. Most of the sample were born in the Republic of Ireland (83.5%), with 6.1% born in the UK and one-in-ten born in a country outside Ireland or the UK (10.4%).

### 5.2 Utilisation of GP services

**Fig. 1:**
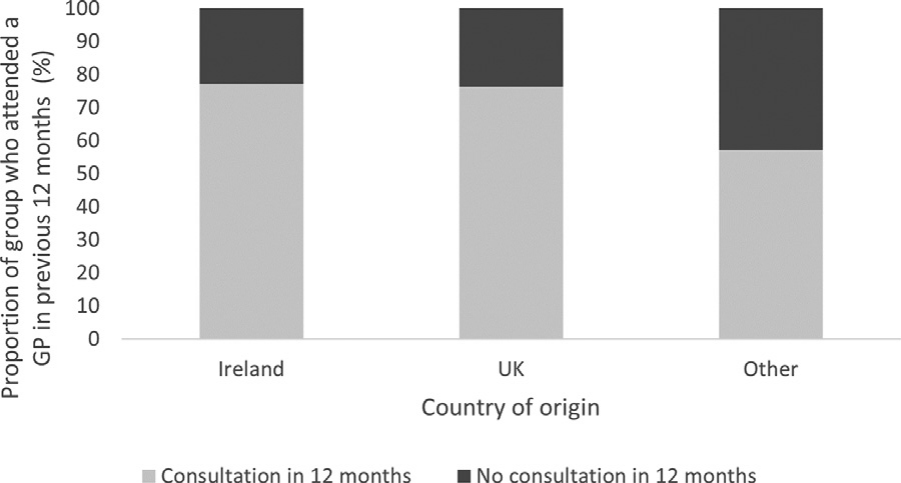
Utilisation of GP services by country of origin.

Fig. 1 indicates a lower rate of utilisation of GP services amongst non-UK immigrants, where amongst the ‘Other’ country of origin group, 57.3% visited the GP in the previous 12 months, compared to 76.4% of those UK-born and 77.3% of native Irish individuals.

The results of the logit regression modelling on the use of GP services presented in Table 2 indicate that there was no statistically significant difference in attendances for GP services between UK-born respondents and those born in Ireland. However, in the fully controlled model (Model 2), non-UK immigrants were less likely (OR 0.62, *p*<0.001) to have visited the GP in the past 12 months than Irish natives.

**Table 2:** Logistic regression results for GP attendance in the previous 12 months, presented as odds ratios.

GP visit in 12 months	Basic model	Full model
Model	(1)	(2)

*Reference: Irish- born*		

UK immigrant	0.948(0.126)[0.730–1.230]	0.915(0.124)[0.701–1.193]

Non-UK immigrant (Other)	0.585***(0.053)[0.490–0.698]	0.615***(0.059)[0.509–0.744]

Male*(Ref: Female)*	0.554***(0.034)	0.562***(0.036)
Age 25–44*(Ref: < 25 years)*	1.023(0.110)	0.943(0.114)
Age 45–64*(Ref: < 25 years)*	1.564***(0.172)	1.071(0.132)
Age 65+*(Ref: < 25 years)*	5.808***(0.772)	1.943***(0.296)
Married*(Ref: Not married)*		1.096(0.078)
Secondary educated*(Ref: Primary education)*		0.921(0.148)
Tertiary educated*(Ref: Primary education)*		0.944(0.160)
Manual labourer*(Ref: Not manual worker)*		0.915(0.088)
Urban*(Ref: Rural)*		0.958(0.097)
GP supply quintile 1 (Low GP provision)*(Ref: Zero GPs in area)*		1.041(0.107)
GP supply quintile 2*(Ref: Zero GPs in area)*		0.984(0.128)
GP supply quintile 3*(Ref: Zero GPs in area)*		0.996(0.135)
GP supply quintile 4*(Ref: Zero GPs in area)*		1.164(0.164)
GP supply quintile 5 (Most GPs)*(Ref: Zero GPs in area)*		1.134(0.170)
Region: Munster*(Ref: Dublin)*		1.045(0.112)
Region: Non-Dublin Leinster*(Ref: Dublin)*		1.326**(0.140)
Region: Connaught-Ulster*(Ref: Dublin)*		1.071(0.125)
Medical card holder*(Ref: No medical or GP card)*		2.188***(0.200)
GP visit card holder*(Ref: No medical or GP card)*		1.571**(0.235)
Private health insurance*(Ref: No private health insurance)*		1.387***(0.107)
Good/very good self-rated health*(Ref: fair/bad/very bad rated-health)*		0.551***(0.078)
Daily smoker*(Ref: Non-smoker/occasional smoker)*		0.773**(0.066)
Long term illness in previous 12 months*(Ref: No reported long-term illness)*		2.719***(0.284)
Diabetes*(Ref: No diabetes)*		1.639(0.475)
Arthritis*(Ref: No arthritis)*		1.274(0.202)
High blood pressure*(Ref: Does not have high blood pressure)*		3.124***(0.490)

Constant	2.686***(0.277)	2.951***(0.766)
N	6326	6326
Log likelihood	−3259.89	−3004.63

Statistical significance indicated by * *p* < 0.05 ** *p* < 0.01 *** *p* < 0.001. Robust standard errors in parentheses. 95% Confidence intervals in square brackets.

Robustness checks available in Tables A2, A3, A4 and A5 of the Supplementary File.

### 5.3 Contact with consultants

**Fig. 2:**
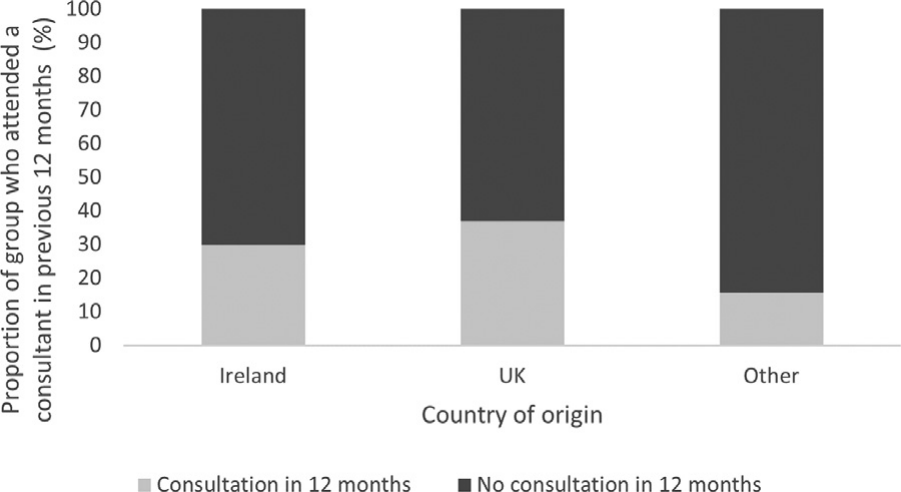
Utilisation of consultant services by country of origin.

Fig. 2 demonstrates that 15.6% of the ‘Other’ country of origin group, representing, non-UK immigrants, attended a consultant in the previous 12 months, compared to 28.8% of native Irish born respondents and 36.8% of UK-born residents. A greater proportion of UK-born respondents attended a consultant than Irish natives.

The results of the logistic regression concerning utilisation of consultant care services by country of birth in Table 3 demonstrates that the UK-born group were more likely to have attended a consultant in the previous 12 months than native respondents (OR: 1.44, *p* = 0.002). On the other hand, non-UK immigrants were less likely than the other two groups to have attended the consultant in the 12 months prior to interview (OR: 0.60, *p*<0.001).

**Table 3:** Logistic regression results for consultant attendance in previous 12 months, odds ratios.

Consultant in 12 months	Basic model	Full model
Model	(1)	(2)

*Reference: Irish- born*		

UK immigrant	1.387**(0.156)[1.112–1.730]	1.438**(0.171)[1.138–1.816]

Non-UK immigrant (Other)	0.590***(0.068)[0.471–0.740]	0.598***(0.074)[0.468–0.763]

Male*(Ref: Female)*	0.764***(0.0438)	0.775***(0.048)
Age 25–44*(Ref: < 25 years)*	1.275(0.167)	0.988(0.142)
Age 45–64*(Ref: < 25 years)*	1.738***(0.225)	1.001(0.144)
Age 65+*(Ref: < 25 years)*	3.227***(0.419)	1.157(0.178)
Married*(Ref: Not married)*		1.284***(0.088)
Secondary educated*(Ref: Primary education)*		0.950(0.107)
Tertiary educated*(Ref: Primary education)*		1.123(0.142)
Manual labourer*(Ref: Not manual worker)*		0.828*(0.079)
Urban*(Ref: Rural)*		1.036(0.104)
GP supply quintile 1 (Low GP provision)*(Ref: Zero GPs in area)*		0.938(0.094)
GP supply quintile 2*(Ref: Zero GPs in area)*		1.069(0.137)
GP supply quintile 3*(Ref: Zero GPs in area)*		1.006(0.134)
GP supply quintile 4*(Ref: Zero GPs in area)*		1.039(0.140)
GP supply quintile 5 (Most GPs)*(Ref: Zero GPs in area)*		1.199(0.171)
Region: Munster*(Ref: Dublin)*		0.631***(0.065)
Region: Non-Dublin Leinster*(Ref: Dublin)*		0.800*(0.079)
Region: Connaught-Ulster*(Ref: Dublin)*		0.722**(0.080)
Medical card holder*(Ref: No medical or GP card)*		1.411***(0.123)
GP visit card holder*(Ref: No medical or GP card)*		1.704***(0.210)
Private health insurance*(Ref: No private health insurance)*		1.541***(0.117)
Good/very good self-rated health*(Ref: fair/bad/very bad rated-health)*		0.475***(0.042)
Daily smoker*(Ref: Non-smoker/occasional smoker)*		0.927(0.084)
Long term illness in previous 12 months*(Ref: No reported long-term illness)*		3.011***(0.224)
Diabetes*(Ref: No diabetes)*		1.216(0.167)
Arthritis*(Ref: No arthritis)*		1.247*(0.121)
High blood pressure*(Ref: Does not have high blood pressure)*		1.174(0.102)

Constant	0.256***(0.031)	0.370***(0.070)
N	6326	6326
Log likelihood	−3651.65	−3283.22

Statistical significance indicated by * *p* < 0.05 ** *p* < 0.01 *** *p* < 0.001. Robust standard errors in parentheses. 95% Confidence intervals in square brackets.

Robustness checks available in Tables A6, A7, A8 and A9 of the Supplementary File.

### 5.4 Robustness check results

The robustness checks performed all indicated that non-UK immigrants were less likely to have used GP care and consultant care in the previous 12 months, as well as in the previous four weeks as presented in the Supplementary File (Tables A2 to A9).

## 6 Discussion

### 6.1 Explaining the results

The empirical analysis suggests that adults residing in Ireland who were born outside Ireland, or the UK were less likely to have used the GP and consultant care in the previous year than the native-born populace. This implies that the rate of utilisation of primary and consultant care amongst immigrants in Ireland is lower than the rest of the population. The forces acting on healthcare utilisation can be framed in terms of the Andersen model which posits that healthcare utilisation is influenced by need, predisposition and enabling factors:

• **Need:** Immigrants in Ireland may be less likely to need healthcare. There is substantial evidence for a healthy immigrant effect in other countries, however, we note that a previous study examining this issue in Ireland found little evidence of a healthy immigrant effect (Nolan 2012). Though we control for self-reported health status and health conditions in this analysis, generally better health may have some residual benefits not captured by these variables.
• **Predisposition:** Immigrants from different cultural backgrounds may have different predispositions and attitudes to healthcare utilisation. In proposing a theoretical framework for immigrant healthcare utilisation, Yang and Hwang (2016, p.7) argue that “there are racial and ethnic differences in healthcare utilisation partly because of genetic predisposition and partly because of culture”. Moreover, there is evidence of differing understandings and valuations of health and when to seek healthcare across different countries. For example, Osipovič (2013) finds that Polish migrants in London delayed seeking treatment or tried to cope with illnesses without contacting formalised medical care, where self-medication and self-care is a first resort. In the Irish context, Kelleher et al. (2020) find that Polish migrants value health more negatively that Irish natives, which the authors hypothesize could result in lower consumption of healthcare services since the perceived benefits of care are less apparent.
• **Enabling factors:**Derose et al. (2007) discussed numerous obstacles which may impede the access of immigrants to healthcare. These included language barriers for immigrants who cannot speak English, socio-economic circumstances and discrimination. Immigrants may also have a lack of health information, lowering their predisposition to go to the GP in their host nation. While medical card status and private health insurance status is controlled for in the analysis, the difficulties of navigating the complex healthcare service in Ireland may be supported by evidence in Figure A1 (included in the Supplementary File) which illustrates that the uninsured rate amongst the non-UK immigrant population is higher than the rest of the sample. Figure A2 (Supplementary File) also demonstrates that immigrants are less likely to be covered by a medical card. In a qualitative investigation, Migge and Gilmartin (2011) concluded that there was a lack of awareness amongst migrants of how to engage with the Irish insurance system and there was considerable concern about the cost of accessing primary care.

### 6.2 Putting the results in the context of the US and European literature

The lower rates of utilisation by non-UK immigrants of primary care doctor services, as well as more specialist consultant physician services in Ireland are akin to findings on this topic from the US (Gil-González et al. 2015). On the other hand, however, UK-born respondents were more likely to use consultant services.

Unlike studies from other European jurisdictions which find a higher use of specialist services by migrants (Norredam et al., 2010), a lower utilisation of consultant doctor services (which are typically in the hospital setting in Ireland) is estimated in this study for the group of non-UK immigrants. The European evidence on the use of GPs is somewhat mixed (Graetz, 2017), though our finding of a lower utilisation of GPs (by non-UK immigrants) is less commonly observed in studies based in European countries. We note the finding on consultant doctor use is consistent then with gatekeeping role of GPs, where if the GP is used less, in turn there is less likelihood of consultant use.

### 6.3 Strengths and limitations

*Healthy Ireland* provides a large cross-sectional sample, representative of the adult population in Ireland. The data contains information on country of birth which permitted the construction of an immigrant status indicator, differentiated by UK and non-UK origin, as well as GP and consultant doctor service use. Additional information on respondents facilitated controlling for a range of key demographic, socioeconomic and health variables in the analysis of the influence of foreign-born background on healthcare utilisation.

Since the dataset is cross-sectional, the work can only comment on the association or link between immigrant group and healthcare utilisation. It cannot make causal claims, though longitudinal data could be used to test this relationship in future studies to derive firmer conclusions. A further limitation of the data is that the sample size of the 'Other', non-UK immigrant group, did not allow for a more detailed breakdown of migrant status by region e.g. Eastern Europe, Western Europe etc. or individual country of birth e.g. Poland or Lithuania. Other unobserved factors which may influence the nature of the relationship between an individual's classification as an immigrant and health also could not be controlled for, for example, length of time of residence in Ireland, attitudes towards the healthcare system, cultural factors etc.

### 6.4 Policy implications

The lower rate of utilisation of healthcare amongst non-UK immigrants in Ireland observed may be a concern from a health policy, health inequalities and healthcare service planning perspective. Informed by guidance from international bodies, the Irish government's intercultural and migrant policies to date recognise issues concerning migrants in using healthcare and provide a statement of commitment for intervention on these, though the results presented here demonstrate that these policies have not proved sufficient solutions. Greater evidence gathering from quantitative and qualitative sources is needed to understand the reasons for the relatively lower utilisation amongst immigrants who were born in countries other than the UK. The evidence presented here may also inform public misconceptions of a perceived burden of immigrants on health and public services.

## 7 Conclusions

The investigation of this paper reveals that immigrants born in countries other than the UK, and residing in Ireland, used GP and consultant-based healthcare at lower levels compared to native-born Irish people. Lower utilisation was not observed for UK-born respondents. This suggests that there may be forces which combine to influence the need, predisposition or access to healthcare amongst some groups of immigrants in Ireland. While the analysis has controlled for many of these predisposing, enabling and need differences between immigrants and natives, the wider literature points to other barriers in terms of discrimination, information gaps, informal networks and language, as well as cultural differences, that cannot be observed directly in the data available here.

The findings suggest that while migrant groups may have relatively lower interactions with the health system in Ireland, they also may be more vulnerable if they are less willing to use healthcare services or have less access to the healthcare system. To facilitate greater integration and prevent health inequalities arising from inequities in access to healthcare between migrants and the native population, further work to understand the reasons for these patterns is required. This work requires input from researchers, policymakers and those engaged in clinical practice.

## Declaration of Competing Interest

The authors declare that they have no known competing financial interests or personal relationships that could have appeared to influence the work reported in this paper.

## Appendix Supplementary materials

Image, application 1

## Footnotes

1 *Healthy Ireland* asks, ‘In which country were you born?’ where a response of ‘the UK’ includes England, Scotland, Wales, Northern Ireland, Isle Of Man, Jersey and Guernsey (Ipsos MRBI 2016b). On the other hand, the 2016 Census of Ireland asks, ‘What Is your place of birth? If Ireland (including Northern Ireland), write in the County. If elsewhere abroad, write in the Country’ (CSO 2016). Thus, in *Healthy Ireland*, those born in Northern Ireland may be included in the UK group, while in the Census those born in Northern Ireland may be considered Irish nationals.
