# Supplementary material for: Utilisation of healthcare by immigrant adults relative to the host population: Evidence from Ireland
Source: J Migr Health. 2021 Nov 26;5:100076. doi: 10.1016/j.jmh.2021.100076 (PMC8715328; doi:10.1016/j.jmh.2021.100076)
Supplement: Supplementary file 1 [file mmc1.docx]

**Utilisation of healthcare by immigrant adults relative to the host population: Evidence from Ireland**

**Supplementary File**

1. Summary statistics of full sample

**Table A1: Summary statistics for Healthy Ireland full sample**

| **Variable** | **Category** | | **Full sample**  **(%)** |
| --- | --- | --- | --- |
| *GP utilisation* | Attended GP in previous 12 months | | 75.8 |
|  | Did not attend GP in previous 12 month | | 24.3 |
| *Consultant utilisation* | Attended consultant in previous 12 months | | 29.1 |
|  | Did not attend consultant in previous 12 months | | 70.9 |
| *Immigrant status* | Irish-born  UK-born  Non-UK born (born in a country outside Ireland and the UK) | | 83.8  5.8  10.4 |
| *Gender* | Male | | 44.3 |
|  | Female | | 55.8 |
| *Age class* | 15-24  25-44  45-64  65 or greater | | 8.1  33.1  32.7  26.1 |
| *Marital status* | Married  Unmarried | | 52.6  47.5 |
| *Education* | Primary | | 11.4 |
|  | Secondary | | 47.3 |
|  | Tertiary | | 41.3 |
| *Social class (manual labourer)* | Yes | | 33.8 |
|  | No | | 66.2 |
| *Urban* | Urban  Rural | | 61.4  38.6 |
| *Region* | Dublin  Munster  Non-Dublin Leinster  Connaught/Ulster | | 22.3  25.8  28.7  23.2 |
| *Supply of GPs in locality (quintile)* | 0.No GP in 1.6km  1.Least GPs in 1.6km  2.  3.  4.  5.Most GPs in 1.6km | | 36.0  15.2  11.9  12.0  13.4  11.6 |
| *Medical card status* | Medical card holder | | 40.4 |
|  | GP visit card holder | | 6.0 |
|  | No medical card or GP visit card | | 53.7 |
| *Private health insurance status* | Has private health insurance  No private health insurance | | 52.4  47.6 |
| *Self-rated health* | Good or very good  Fair, poor or very poor | | 81.9  18.0 |
| *Smoker* | Yes  No | | 17.3  82.7 |
| *Long term Illness in past 12 months* | Yes  No | | 31.5  68.5 |
| *Diabetes* | Yes  No | | 5.3  94.7 |
| *Arthritis* | Yes  No | | 13.1  86.9 |
| *High blood pressure* | Yes  No | | 15.8  84.2 |
| Sample observations |  | 7,498* | |

2. Utilisation of General Practitioner services

**Table A2: Odds ratio (OR) of immigrant group on use of GP services using proportional partial odds model**

| **Base category: No visit to GP in previous 12 months** | **Basic model** | **Full model** |
| --- | --- | --- |
|  | **(1)** | **(2)** |
| **1. Odds of having visited GP in the past 12 months but not the last 4 weeks** | | |
| Reference: Irish born |  |  |
|  |  |  |
| UK immigrant | 0.935  (0.094) | 0.891  (0.091) |
|  |  |  |
| Non-UK immigrant | 0.567***  (0.050) | 0.595***  (0.056) |
| **2. Odds of having visited GP in the previous 4 weeks** | | |
|  |  |  |
| UK immigrant | 0.935  (0.094) | 0.891  (0.091) |
|  |  |  |
| Non-UK immigrant | 0.812  (0.089) | 0.795*  (0.092) |
| Observations (N) | 6,326 | 6,326 |
| Log likelihood | -6338.59 | -5910.38 |
| Statistical significance indicated by * p < 0.05 ** p < 0.01 *** p < 0.001. Robust standard errors in parentheses.  Model (1) adjusts for immigrant group (reference: Irish-born), gender, age.  Full model, Model (2) is Model (1) with additional controlling for medical card status, insurance status, marital status, unskilled labourer, region, level of education, urbanity, whether they smoke, illness in past 12 months, self-rated health, diabetes, arthritis, whether they have high blood pressure. Full results available on request from the author. | | |

**Table A3: OLS regression of GP utilisation in previous 4 weeks**

|  | **Basic model** | **Full model** |
| --- | --- | --- |
| **Model** | **(1)** | **(2)** |
| Reference: Irish born |  |  |
|  |  |  |
| UK immigrant | -0.013  (0.034) | -0.028  (0.032) |
|  |  |  |
| Non-UK immigrant | -0.060**  (0.023) | -0.060**  (0.023) |
|  |  |  |
| N | 6,326 | 6,326 |
| R^2^ | 0.03 | 0.14 |
| Statistical significance indicated by * p < 0.05 ** p < 0.01 *** p < 0.001. Robust standard errors in parentheses.  Model (1) adjusts for immigrant group (reference: Irish-born), gender, age.  Full model, Model (2) is Model (1) with additional controlling for medical card status, insurance status, marital status, unskilled labourer, region, level of education, urbanity, whether they smoke, illness in past 12 months, self-rated health, diabetes, arthritis, whether they have high blood pressure. Full results available on request from the author. | | |

**Table A4: OR for ordered logistic regression of GP utilisation in previous 12 months**

|  | **Basic model** | **Full model** |
| --- | --- | --- |
| **Model** | **(1)** | **(2)** |
| Reference: Irish born |  |  |
|  |  |  |
| UK immigrant | 0.938  (0.093) | 0.893  (0.089) |
|  |  |  |
| Non-UK immigrant | 0.626***  (0.056) | 0.634***  (0.059) |
| N | 6,326 | 6,326 |
| Log likelihood | -7615.40 | -5999.95 |
| Statistical significance indicated by * p < 0.05 ** p < 0.01 *** p < 0.001. Robust standard errors in parentheses.  Model (1) adjusts for immigrant group (reference: Irish-born), gender, age.  Full model, Model (2) is Model (1) with additional controlling for medical card status, insurance status, marital status, unskilled labourer, region, level of education, urbanity, whether they smoke, illness in past 12 months, self-rated health, diabetes, arthritis, whether they have high blood pressure. Full results available on request from the author. | | |

**Table A5: OR of logistic regression model of GP visit in previous 12 months, where participants**

**with missing observations on included variables were not dropped**

|  | **Basic model** | **Full model** |
| --- | --- | --- |
| **Model** | **(1)** | **(2)** |
| Reference: Irish born |  |  |
| UK immigrant | 0.930  (0.116) | 0.938  (0.122) |
|  |  |  |
| Non-UK immigrant | 0.599***  (0.050) | 0.638***  (0.057) |
| N | 7,498 | 7,436 |
| Log likelihood | -3823.77 | -3459.31 |
| Statistical significance indicated by * p < 0.05 ** p < 0.01 *** p < 0.001. Robust standard errors in parentheses.  Model (1) adjusts for immigrant group (reference: Irish-born), gender, age.  Full model, Model (2) is Model (1) with additional controlling for medical card status, insurance status, marital status, unskilled labourer, region, level of education, urbanity, whether they smoke, illness in past 12 months, self-rated health, diabetes, arthritis, whether they have high blood pressure. Full results available on request from the author. | | |

3. Utilisation of Consultant services

**Table A6: OR of utilisation of Consultant care employing the partial proportional odds model**

| **Base category: No visit to consultant in previous 12 months** | **Basic model** | **Full model** |  |
| --- | --- | --- | --- |
| **Model** | **(1)** | **(2)** |  |
| **1. Odds of having visited consultant in the past 12 months but not the last 4 weeks** | | |  |
| Reference: Irish born |  |  |  |
|  |  |  |  |
| UK immigrant | 1.334***  (0.141) | 1.471  (0.176) |  |
|  |  |  |  |
| Non-UK immigrant | 0.598***  (0.070) | 0.617***  (0.077) |  |
| **2. Odds of having visited consultant in the previous 4 weeks** | | |  |
|  |  |  |  |
| UK immigrant | 1.334***  (0.141) | 1.017  (0.189) |  |
|  |  |  |  |
| Non-UK immigrant | 0.598***  (0.070) | 0.617***  (0.077) |  |
| Observations (N) | 6,314 | 6,314 |  |
| Log likelihood | -4744.95 | -4350.25 |  |
| Statistical significance indicated by * p < 0.05 ** p < 0.01 *** p < 0.001. Robust standard errors in parentheses.  Model (1) adjusts for immigrant group (reference: Irish-born), gender, age.  Full model, Model (2) is Model (1) with additional controlling for medical card status, insurance status, marital status, unskilled labourer, region, level of education, urbanity, whether they smoke, illness in past 12 months, self-rated health, diabetes, arthritis, whether they have high blood pressure. Full results available on request from the author. | | |  |

**Table A7: OLS regression on the utilisation of consultant care in the previous 4 weeks**

|  | **Basic model** | **Full model** |
| --- | --- | --- |
| **Model** | **(1)** | **(2)** |
| Reference: Irish born |  |  |
|  |  |  |
| UK immigrant | -0.030  (0.025) | -0.025  (0.019) |
|  |  |  |
| Non-UK immigrant | -0.037**  (0.012) | -0.059*  (0.028) |
|  |  |  |
| N | 6,326 | 6,326 |
| R^2^ | 0.003 | 0.17 |
| Statistical significance indicated by * p < 0.05 ** p < 0.01 *** p < 0.001. Robust standard errors in parentheses.  Model (1) adjusts for immigrant group (reference: Irish-born), gender, age.  Full model, Model (2) is Model (1) with additional controlling for medical card status, insurance status, marital status, unskilled labourer, region, level of education, urbanity, whether they smoke, illness in past 12 months, self-rated health, diabetes, arthritis, whether they have high blood pressure. Full results available on request from the author. | | |

**Table A8: OR of ordered logistic regression estimates on had a consultant visit in previous 4 weeks and previous 12 months**

|  | **Basic model** | **Full model** |
| --- | --- | --- |
| **Model** | **(1)** | **(2)** |
| Reference: Irish born |  |  |
|  |  |  |
| UK immigrant | 1.334**  (0.141) | 1.373**  (0.150) |
|  |  |  |
| Non-UK immigrant | 0.598***  (0.070) | 0.615***  (0.077) |
| N | 6,314 | 6,314 |
| Log likelihood | -5666.91 | -4355.86 |
| Statistical significance indicated by * p < 0.05 ** p < 0.01 *** p < 0.001. Robust standard errors in parentheses.  Model (1) adjusts for immigrant group (reference: Irish-born), gender, age.  Full model, Model (2) is Model (1) with additional controlling for medical card status, insurance status, marital status, unskilled labourer, region, level of education, urbanity, whether they smoke, illness in past 12 months, self-rated health, diabetes, arthritis, whether they have high blood pressure. Full results available on request from the author. | | |

**Table A9: OR of logistic regression on had a consultant attendance in previous 12 months, where participants with missing observations on included variables were not dropped**

|  | **Basic model** | **Full model** |
| --- | --- | --- |
| **Model** | **(1)** | **(2)** |
| Reference: Irish born |  |  |
| UK immigrant | 1.340***  (0.142) | 1.369***  (0.154) |
|  |  |  |
| Non-UK immigrant | 0.603***  (0.063) | 0.615***  (0.069) |
|  |  |  |
| N | 7,498 | 7,356 |
| Log likelihood | -4361.38 | -3894.11 |
| Statistical significance indicated by * p < 0.05 ** p < 0.01 *** p < 0.001. Robust standard errors in parentheses.  Model (1) adjusts for immigrant group (reference: Irish-born), gender, age.  Full model, Model (2) is Model (1) with additional controlling for medical card status, insurance status, marital status, unskilled labourer, region, level of education, urbanity, whether they smoke, illness in past 12 months, self-rated health, diabetes, arthritis, whether they have high blood pressure. Full results available on request from the author. | | |

3. Graphs of indicating levels of healthcare access across country of origin

**Figure A1: Private health insurance by country of origin**

**Figure A2: Medical card status by country of origin**
